# The use of whole-genome sequencing and development of bioinformatics to monitor overlapping outbreaks of *Candida auris* in southern Nevada

**DOI:** 10.3389/fpubh.2023.1198189

**Published:** 2023-07-13

**Authors:** Andrew Gorzalski, Frank J. Ambrosio, Lauryn Massic, Michelle R. Scribner, Danielle Denise Siao, Chi Hua, Phillip Dykema, Emily Schneider, Chidinma Njoku, Kevin Libuit, Joel R. Sevinsky, Stephanie Van Hooser, Mark Pandori, David Hess

**Affiliations:** ^1^Nevada State Public Health Laboratory, Reno, NV, United States; ^2^Theiagen Consulting LLC, Highlands Ranch, CO, United States; ^3^Division of Disease Control and Health Statistics, Washington State Department of Health, Public Health Laboratories, Shoreline, WA, United States; ^4^Nevada Department of Health and Human Services, Las Vegas, NV, United States; ^5^Department of Pathology and Laboratory Medicine, University of Nevada, Reno School of Medicine, Reno, NV, United States; ^6^Department of Microbiology and Immunology, University of Nevada, Reno School of Medicine, Reno, NV, United States

**Keywords:** *Candida auris*, epidemiology, whole-genome sequencing, bioinformatics, emerging pathogens

## Abstract

A *Candida auris* outbreak has been ongoing in Southern Nevada since August 2021. In this manuscript we describe the sequencing of over 200 *C. auris* isolates from patients at several facilities. Genetically distinct subgroups of *C. auris* were detected from Clade I (3 distinct lineages) and III (1 lineage). Open-source bioinformatic tools were developed and implemented to aid in the epidemiological investigation. The work herein compares three methods for *C. auris* whole genome analysis: Nullarbor, MycoSNP and a new pipeline TheiaEuk. We also describe a novel analysis method focused on elucidating phylogenetic linkages between isolates within an ongoing outbreak. Moreover, this study places the ongoing outbreaks in a global context utilizing existing sequences provided worldwide. Lastly, we describe how the generated results were communicated to the epidemiologists and infection control to generate public health interventions.

## 1. Introduction

*Candida auris* was first identified in 2009 in Japan and has quickly become an emerging global pathogen (1). Since its discovery it has rapidly spread worldwide (2–4). Genomic analysis of *C. auris* has identified four main clades in addition to a rarer fifth clade (5). *C. auris* clades were initially identified by geographical region—Clade 1 (South Asian), Clade 2 (East Asian), Clade 3 (African), Clade 4 (South American) and Clade 5 (Middle Eastern) (2, 5). However, outside of Clade 5, all other clades have escaped their initial geographic boundaries (6).

*C. auris* presents multiple medical and public health challenges which contribute to its concern as an emerging pathogen. Firstly, *C. auris* commonly possesses resistance to existing antifungal pharmaceuticals (2). Secondly, *C. auris* has the ability to colonize hosts both internally and externally and often asymptomatically. This facilitates spread (6, 7) and obfuscates screening strategies. Thirdly, these traits facilitate establishment and spread within health care facilities which has prompted agencies around the world (Centers for Disease Control [CDC], European Centre for Disease Prevention and Control [EDPC] and Public Health England) to release clinical alerts on *C. auris* (8–10). Fourthly, crude estimates of mortality for hospitalized patients with candidemia is 30–72% in frequently hospitalized indivivduals.

Because of the ability of *C. auris* to spread and colonize in health care facilities, rapid identification and genomic analysis are necessities in containing outbreaks. In this study we applied robust genomic sequencing analysis to a major outbreak of *C. auris* in southern Nevada. Analysis revealed two genomically distinct, simultaneous *C. auris* outbreaks that initiated with chronological proximity. Whole genome sequencing was performed on 208 isolates associated with the outbreak. The sequences generated were utilized both to develop and to assess novel tools. These tools were utilized for identification and for phylogenetic analysis to aid the epidemiologic investigation. Three existing methodologies for analyzing *C. auris* whole genome sequences were studied and the results are shown: Nullarbor (12, 13), mycosnp (3) and TheiaEuk (14). All methods showed the ability to identify *C. auris* based on whole genome sequencing and to generate relatedness metrics. Using these tools, we describe the development of a custom, shared single-nucleotide polymorphism (SNP) method that may provide significant aid in the use of *C. auris* genomic sequences in epidemiologic investigations.

## 2. Materials and methods

### 2.1. Collection of specimens

Specimens were isolated from clinical samples collected in Nevada from August 2021 to July 2022. Two additional isolates of interest from Nevada are included in this study from 2022-11-18 and 2023-01-15. *C. auris* is not a reportable organism in Nevada, so initial clinical samples were obtained in collaboration with our ARLN lab in Washington State and southern Nevada clinical partners. Since, *C. auris* is not a reportable organism, so it is difficult to estimate the number of cases compared to the number sequenced in this timeframe. However, this study sequenced every *C. auris* isolate from the time range noted that the Nevada State Public Health Laboratory was able to obtain a cultured isolate. All relevant information for each *C. auris* isolates including clade designation, Sequence Read Archive identifier at NCBI, antifungal MICs, etc. is included in Supplementary Table S1.

### 2.2. Whole-genome sequencing of *Candida auris*

Genomic DNA used for sequencing was extracted using a combination of bead-beating (FastPrep-24, MP Biomedicals, Irvine, CA) and magnetic-bead purification (Maxwell RSC 48, Promega, Madison, WI). First, isolates from Sabouraud Dextrose agar plates were mixed with silica beads (Lysing Matrix C, MP Biomedical) and then mechanically sheared with 2 cycles at 6.0 m/s for 30 s with a 5 min pause between (FastPrep-24, MP Biomedical). Genomic DNA was extracted using PureFood Pathogen Kit (Promega) on a Maxwell RSC 48 (Promega) using manufacturer’s protocol. Genomic DNA was library prepped using DNA Prep Kit (Illumina, San Diego, CA) using manufacturer’s recommended protocol using a STARlet automated liquid handler (Hamilton Company, Reno, NV). Paired-end sequencing (2×151) was performed using Illumina’s MiniSeq and NovaSeq 6,000 to a minimum depth of 35x average coverage.

### 2.3. Antibiotic susceptibility testing

*Candida auris* AST was performed using microbroth dilution and predefined gradient of antibiotic concentrations (Etest) methods. A patient isolate was grown on Sabouraud Dextrose agar plate and incubated at 30°C in ambient air for 24 h and used to make 0.5 McFarland inoculum suspension in demineralized sterile water. The 0.5 McFarland suspension was measured by spectrophotometer to verify the 0.5 McFarland (80–82% transmittance). Twenty microliters of 0.5 McFarland suspension were added into 11 mL of RPMI broth tube, and 100 μL of the RPMI diluted sample was distributed to each well of a 96-well plate pre-loaded with antibiotics and incubated along with control plates for 24 h at 35°C. The same 0.5 McFarland inoculum suspension was used to inoculate a RPMI agar plate using a sterile cotton swab. A single Amphotericin B Etest strip was applied to middle of the agar surface using sterile forceps and incubated along with control plates for 24 h at 35°C. The AST of the microbroth dilution panel was read using parabolic magnifying mirror to determine the MIC (lowest concentration where there is ≤50% growth compared to growth control well). For the Amphotericin B Etest, MIC was interpreted at value where there is 100% growth inhibition (number above where ellipse intercepts Etest strip).

### 2.4. Nullarbor implementation

Nullarbor is a reads-to-report bioinformatics pipeline originally written in Perl. In the Terra workflow version, Nullarbor is implemented as a single task using the Workflow Description Language (WDL). Reads are accepted in two separate arrays for read file one and read file two (n= 16). A tsv input file is generated by iterating through the arrays of read files, and this sample sheet tsv is ultimately passed into the Nullarbor analysis module. Additional inputs include an array of sample names, and a reference genome. The clade specific reference genome should be used, meaning clade must be discerned prior to running this workflow, as there is no clade typing module.

Read cleaning is performed removing sequencing adaptors and low-quality input sequencing data using Trimmomatic (15). Species identification is performed using Kraken 2 with the EuPathDB64 database available here^1^ (16). *De novo* assembly is performed using SKESA (17). In addition, sequencing reads are aligned to a user-provided reference genome using Snippy, and the core phylogeny and SNP matrix are produced using snippy-core (18).

### 2.5. MycoSNP implementation

MycoSNP is an open-source bioinformatics pipeline designed to call variants and construct a phylogeny from mycotic pathogen next generation sequencing data (3). The original version of this tool was written in Nextflow and implemented by the CDC Mycotics Disease Branch^2^ (19). The components of this tool are wrapped in docker containers. Each of these components is an established bioinformatics method, and output files are in standard format so as to allow compatibility with downstream analytical modules. The inputs to this workflow include the raw read FASTQ files from an Illumina paired end sequencing run and a reference genome in FASTA file format. The reference genomes utilized were the CDC clade 1 reference genome [strain B11205] (GenBank Accession GCA_016772135.1) and the CDC clade 3 reference genome [strain B11221] (GenBank Accession GCA_002775015.1) (5).

MycoSNP was run with default settings as described by Bagal et al (3). The first step of the pipeline is to prepare the reference genome for alignment by masking repeat regions using nucmer^3^ and generating an index for efficient alignment with the Burrows-Wheeler Aligner (BWA).^4^ Next, the FASTQ files are processed and checked for quality. For FASTQ processing, SeqKit^5^ is used to filter unpaired reads, SeqTK^6^ is used to downsample reads, and FaQCs^7^ is used to perform quality checks and read trimming. After processing, the reads are aligned to the reference genome using BWA. The resulting binary alignment map (BAM) files are sorted with SAMTools^8^ and processed to remove duplicates, ensure mate-paired read information is correct, and add read groups with Picard.^9^ This final step of the alignment process is to perform additional quality checks using FastQC^10^ and MultiQC.^11^ Variants are called using GATK.^12^ The resulting GVCF files from each sample are then combined into a single VCF file, which is then filtered based on normalized variant quality, Phred-scaled probability of strand bias, mapping quality of all reads at the variant site, and the number of filtered reads that support each of the alleles found at the variant site.^13^ The combined and filtered VCF is then split into individual sample-specific VCF files. Using BCFTools^14^ and SeqTK, a consensus sequence is generated for each sample, and these sequences are combined into a multi-FASTA to be used as the input to the phylogenetic tree construction tools.

Multiple phylogenies are generated in MycoSNP. The phylogenetic inference tools rapidNJ,^15^ FastTree2,^16^ RaxML,^17^ and IQTree^18^ are all utilized in this final step of the workflow.

To make this workflow available on the Terra platform, the original pipeline has been split into two separate tools, each wrapped in a WDL workflow. The two new workflows perform variant calling and phylogenetic analysis independently, but the underlying components are the same as the original MycoSNP.

### 2.6. TheiaEuk implementation

The TheiaEuk_PE workflow performs the assembly, quality assessment, and genomic characterization of fungal genomes (14). This cloud-native workflow is implemented in the Workflow Description Language and has been operationalized on the Terra.bio platform. TheiaEuk_PE has been fashioned to accept Illumina paired-end sequencing data as the primary input but offers many optional inputs to allow the user to specify parameters for all internal components of the workflow. Input reads are preprocessed with a raw-read quality assessment followed by read cleaning (quality trimming and adapter removal), and then an additional quality assessment of the cleaned reads. Subsequently, *de novo* assembly is performed using the Shovill package with SKESA set as the default assembler. SKESA is implemented using default parameters. Once the assembly has been generated an assembly quality assessment is performed using QUAST. Using the assembly, species taxon identification is performed by GAMBIT (20). The GAMBIT implementation in TheiaEuk_PE uses a custom fungal database containing 5,667 genomes and 245 species. For some taxa identified, taxa-specific sub-workflows will be automatically activated, launching additional taxa-specific characterization tools, including a GAMBIT-based clade-typing tool and antifungal resistance detection performed using Snippy variant calling with a custom query for genes in which there are known antifungal-resistance conferring mutations (14). For *C. auris*, TheiaEuk queries the Snippy results for strings matching the *FKS1*, *ERG11*, and *FUR1* genes.

### 2.7. Benchmarking against other workflows

Three workflows were compared in this study using two sets of *C. auris* reads. The first set was 60 samples from clade 1 and the second set was 148 samples from clade 3. TheiaEuk was combined with kSNP3 to produce phylogenetic trees and SNP matrices. First, TheiaEuk was used to produce assemblies which were then used as inputs to the kSNP3 workflow to produce a pair of phylogenetic trees and SNP matrices. MycoSNP_Variants was used to produce VCF files which were fed into the MycoSNP_Tree workflow to produce a set of phylogenetic trees and a SNP matrix. Nullarbor was run as a single workflow producing a SNP matrix and a phylogenetic tree. Each VM deployed to run these workflows was given runtime parameters of 32 cpus and 128 GB of memory. These compute resources were allocated to each VM, so workflows that launched several VMs simultaneously took advantage of parallelization.

### 2.8. *Candida auris* specific subroutines within TheiaEuk

Upon the taxonomic assignment of *C. auris* to a sample, TheiaEuk_PE automatically triggers two taxa-specific sub-workflows (14). First, a clade-typing workflow is launched. Clade-typing is performed using a modified version of the GAMBIT module to determine which of the five clade specific references most closely matches the query sequence. The output of the clade-typing module includes the clade assignment as well as a clade-specific annotated genome which is then passed to the antifungal resistance detection module. Snippy is used to align reads to the annotated reference genome and call variants. The variants are annotated with the genes in which they are found because the input reference genome is annotated. The variants are then queried for any that occur within genes known to contain resistance conferring mutations. This method is used rather than reporting only known resistance conferring mutations to ensure that novel resistance conferring mutations are not ignored.

### 2.9. Shared SNP analysis

This analysis uses the VCF file from kSNP3 which lists each unique SNP in a dataset with the 9 base pairs upstream and downstream of the SNP location (21). The SNP output for clade 1 is made against the CDC clade 1 reference genome [strain B11205] (GenBank Accession GCA_016772135.1) and the SNP output for clade 3 is made against the CDC clade 3 reference genome [strain B11221] (GenBank Accession GCA_002775015.1) (5). The VCF file displays if each SNP is present, absent or unassembled for each input genomes. This analysis focuses only on SNPs that are assembled in each input genome and then filters out from that group SNPs that are in every input genome (save the reference genome) and SNPs that are unique to only one of the input genomes. The SNPs that remain are referred to as “shared SNPs,” falling somewhere between unique and present in all genomes in your query set. These SNPs are manually clustered to form groups that have unique patterns of shared SNPs. These SNPs were not annotated in our analysis.

## 3. Results

### 3.1. NV outbreak

An outbreak of *C. auris* in southern Nevada was first detected in August 2021. As of October 31st, 2022, over 500 cases had been reported with over 200 isolates preserved. We report on 210 isolates including 2 isolates that represent new introductions to Nevada as detected by our analysis pipelines described below. All isolates were subjected to whole genome sequencing.

### 3.2. Global relatedness of southern Nevada clades to global clades

**Figure 1:**
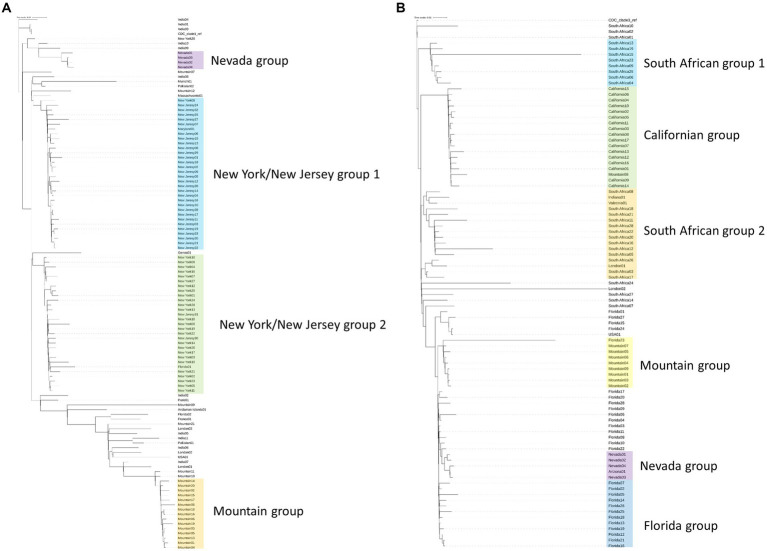
Phylogenetic trees generated by kSNP3 (21) on *Candida auris* isolates. Isolates are labeled by region from which they were isolated. Samples for each tree are listed with their SRA ID in Supplemental Table 2. Shading on the tree represents highly related branches which clustered by geography. **(A)** Phylogenetic tree of Clade 1 isolates including 4 isolates from the southern Nevada outbreak (colored in purple). **(B)** Phylogenetic tree of Clade 3 isolates including 5 isolates from the southern Nevada outbreak (colored in purple).

To initially assess isolates associated with the outbreak genomically, we utilized a phylogenetic tree-based comparison on the entire genome against a subsampling of previously submitted clade 1 or clade 3 strains from organizations around the world (Figures 1A,B; Supplementary Table S2) (21). These trees establish that the southern Nevada strains have a unique phylogenetic signature among all *C. auris* isolates previously submitted to public repositories. Figure 1A presents *C. auris* clade 1 samples that have been sequenced and uploaded to public repositories. The major phylogenetic groups are highlighted with different colors and annotated by the region where the *C. auris* isolates were collected with the Nevada isolates highlighted in purple. The Nevada clade 1 outbreak is genetically distinct from other outbreaks in the U.S. as shown in Figure 1A. The index southern Nevada case for the clade 1 outbreak was SRR23249008 (Supplementary Table S1).

A similar analysis was performed with clade 3 samples and is shown in Figure 1B. As with clade 1 analysis, it became clear that the Nevada clade 3 samples (highlighted in purple) were genetically distinct from other previously sequenced outbreaks. The index southern Nevada cases for the clade 3 outbreak were SRR19738700 and SRR23109087 which were both collected on 11-02-2021 in the case facility (Supplementary Table S1). Note the one isolate labeled Arizona01 in the shaded purple box was collected in Arizona from a southern Nevadan patient. Epidemiological investigation strongly suggested the patient contracted *C. auris* in southern Nevada prior to travel to Arizona (data not shown). The information on the case described in the previous sentence was obtained and shared with the Nevada State Public Health Laboratory in collaboration with our public health partners in Utah and Nevada. These data were collected and shared in accordance with IRB protocols. We concluded having a pipeline(s) of rigorous bioinformatic tools capable of handling fungal microbes would be necessary for the public health response to these simultaneous and distinct outbreaks occurring in southern Nevada.

### 3.3. State of fungal bioinformatic whole-genome sequencing pipelines *circa* march 2022

After an initial analysis of the outbreak specimens with regard to clade, we sought to further determine the utility of phylogenetic analysis to assist disease control. Upon initiation of sequencing and genomic analysis of the outbreak at the Nevada State Public Health Laboratory, one computational method /pipeline was available for assessments of *C. auris* (12, 13). Another was completed but unpublished (3). Difficulties with implementation of the former led our group to develop a novel pipeline for identification and phylogenetic analysis of *C. auris* genomes. This pipeline has been named “TheiaEuk” (14). As we sought to determine the best methods for using sequencing to assist disease control efforts for this outbreak, we sought to compare all three methods in terms of capability and functionality.

### 3.4. Pipeline comparisons for *Candida auris* genome assemblies

**Figure 2:**
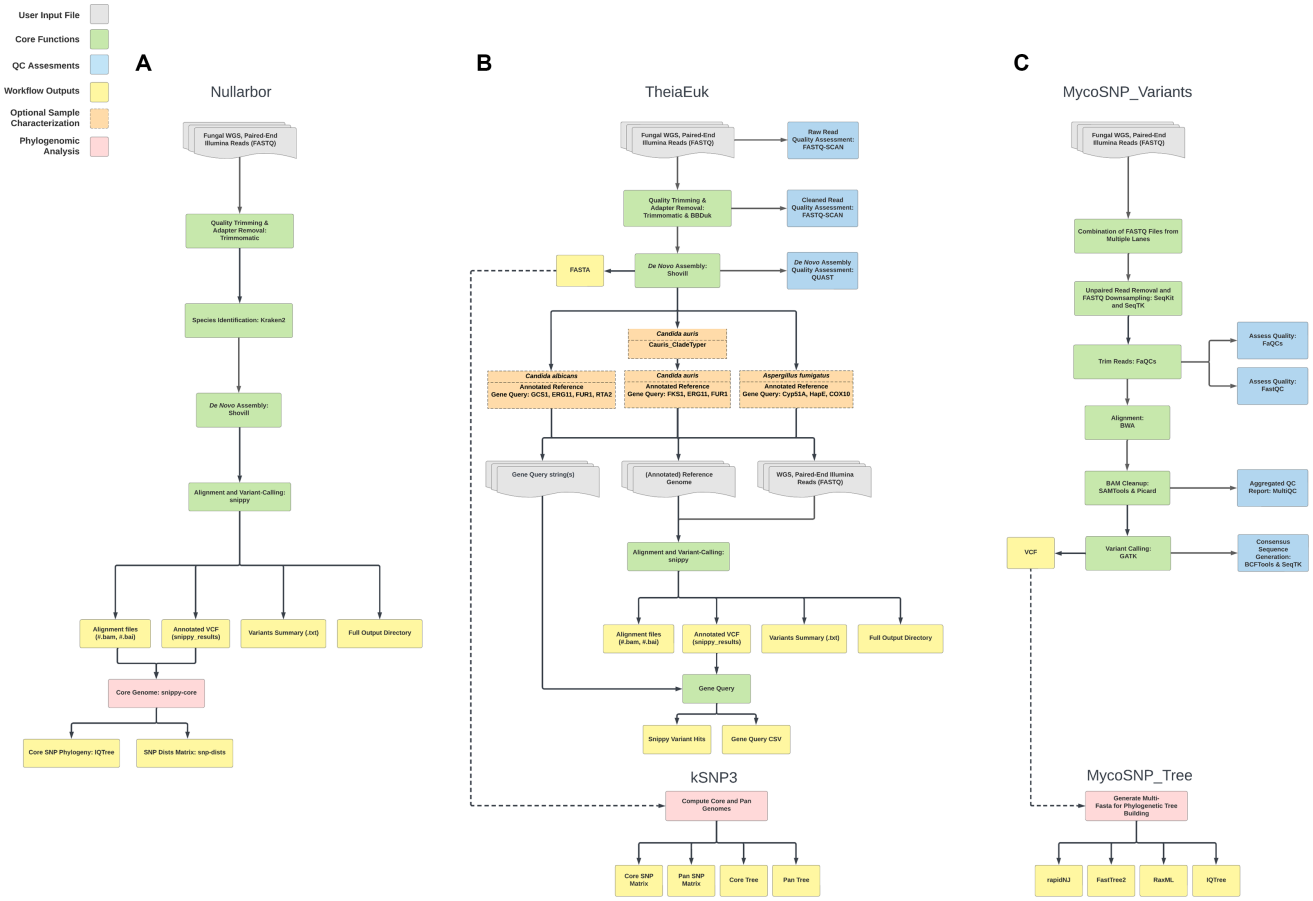
Workflow comparisons of whole-genome sequencing bioinformatic pipelines that analyze *C. auris* WGS data. The shaded key highlights the major steps performed in analysis for ease of comparison. For details on each workflow see Materials and Methods. **(A)** Nullarbor **(B)** TheiaEuk **(C)** MycoSNP_variants.

In testing three methods, we assembled genomes (*n* = 60) from clade I from patients found infected by *C. auris* in southern Nevada. Assembly and downstream analyses were completed using each of three workflows: Nullarbor, TheiaEuk and Mycosnp. Upon completion, results from Nullarbor needed no additional analysis. TheiaEuk and Mycosnp required additional step for the generation of SNP matrices and/or phylogenetic trees (Figure 2). Times required for analyses are shown in Table 1. As shown in Table 2, all methods assemble genomes of nearly identical sizes with the average genome lengths of Nullarbor being 12,276,509 bp, TheiaEuk being 12,288,829 bp and MycoSnp being 12,406,106 bp. For assembly, Nullarbor uses Shovill v1.1.0 with SKESA v2.4.0 as the default setting. TheiaEuk uses Shovill v1.1.0 with SKESA v2.4.0 as the default setting. MycoSNP uses a reference guided assembly that produces the same genome length for each sample using method BWA v0.7.17 for read alignment and GATK v4.2.5.0 for variant calling. MycoSNP using reference guided assembly creates a single contig per chromosome where Nullarbor produces an average of 683 contigs from our Clade I samples and TheiaEuk produces an average of 505 contigs from tested clade I samples (Table 2).

**Table 1:** Run times for each of the tested WGS pipelines on both the Clade 1 and Clade 3 isolate sets.

	Clade 1 [time (hr:min)]	Clade3 [time (hr:min)]
TheiaEuk (Average)	2:25	2:47
kSNP3	1:09	3:03
Total	3:34	5:50
MycoSNP (Average)	7:09	8:19
MycoSNP_Tree	0:24	1:11
Total	7:33	9:30
Nullarbor	25:16:00	53:44:00

For Clade 1 *n* = 60 and for Clade 3 *n* = 148.

**Table 2:** For the Clade 1 dataset (*n* = 60) the comparison of WGS assembly stats (genome length and number of contigs) is reported.

	Mean genome size (bps)	Number of contigs
Nullarbor	12,276,509 (± 27,775)	683 (± 215)
TheiaEuk	12,288,829 (± 25,062)	505 (± 248)
MycoSNP	12,406,106	NA

### 3.5. Benchmarking TheiaEuk, MycoSNP and Nullarbor

Comparison of the three whole-genome sequencing pipelines based on analysis time was performed on the same test set described in the previous section (Table 1). All pipelines were run with the same virtual machines (Materials and Methods). MycoSNP had the fastest report time at 2 h and 5 min, followed by TheiaEuk at 8 h and 10 min with nullarbor requiring 26 h and 12 min.

### 3.6. Pipeline comparisons for *Candida auris* SNP matrices

Each method produces SNP matrices which display the calculated number of SNPs between each sample in an analyzed set. We compared the number of SNPs detected by each method compared to the first clade I sample by numerical order based on our internal nomenclature (SRR19664611) to all other samples. We then calculated the absolute differences between each pairwise SNP comparison between two methods. Comparing Nullarbor and TheiaEuk the difference was 1.9 (± 2.1) SNPs with Nullarbor consistently reporting fewer SNPs. Comparing Nullarbor and MycoSNP the difference was 1.9 (± 2.2) SNPs with Nullarbor consistently reported fewer SNPs. Comparison of TheiaEuk and MycoSNP resulted in a difference of 0.57 (± 0.89) SNPs with MycoSNP consistently reporting more SNPs (Table 3).

**Table 3:** Calculated SNP differences between the WGS pipelines for Clade 1 (*n* = 60).

Assembly Method #1	Assembly Method #2	Ave Difference in SNP Matrix (± SNPs)
MycoSNP	TheiaEuk	0.57 (± 0.89)
MycoSNP	Nullarbor	1.9 (± 2.2)
TheiaEuk	Nullarbor	1.9 (± 2.1)

### 3.7. Development of a pipeline to distinguish fine grain differences in ongoing outbreaks

**Figure 3:**
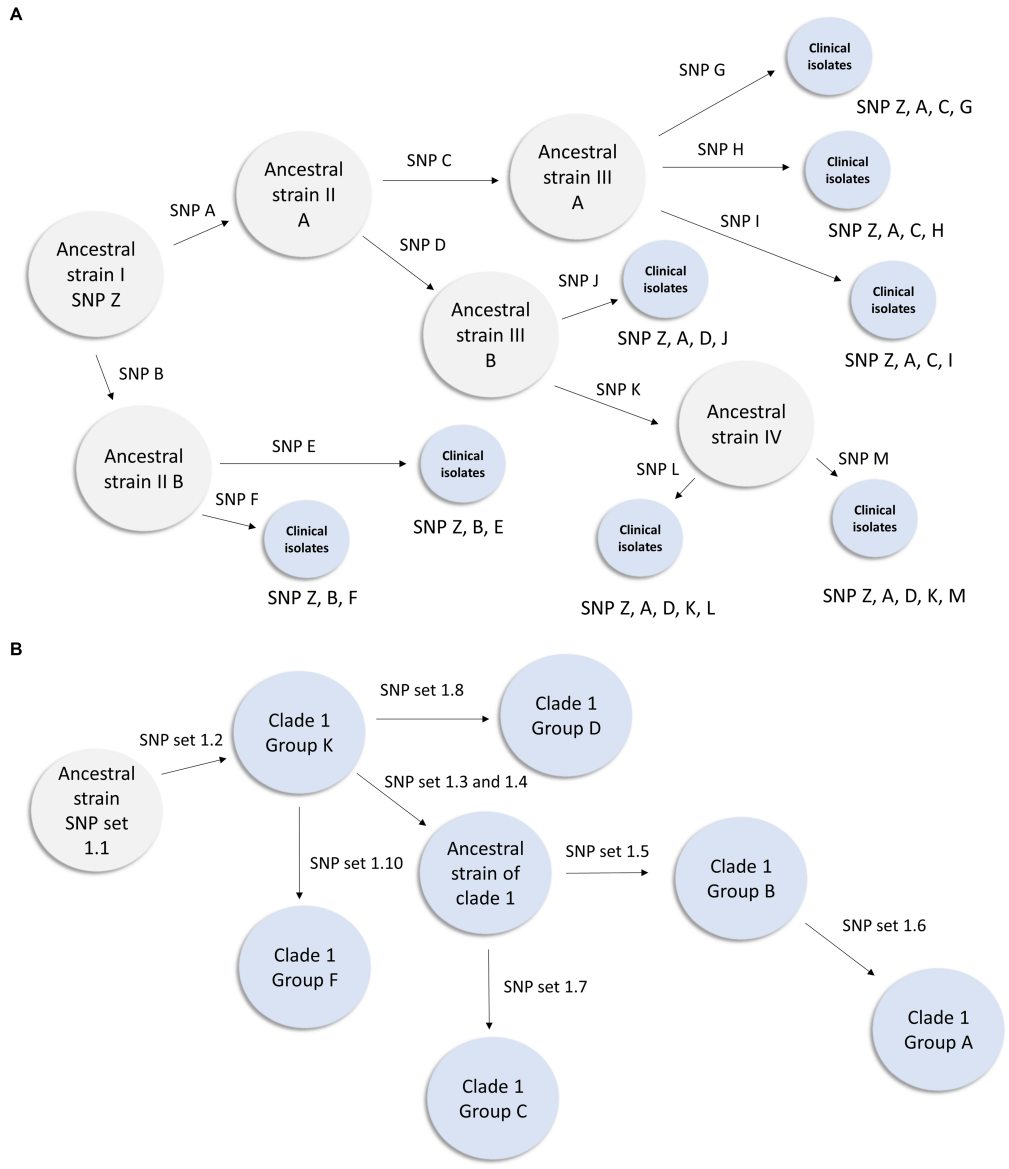
Inferred transmission networks based on shared SNP analysis. **(A)** Figure illustrates how theoretical Ancestral strain I (with SNP Z) could evolve during an outbreak. When an isolate acquires a novel SNP or SNPs during this hypothetical outbreak, an arrow is displayed and a designation (such as SNP A) is shown above the arrow. Gray circles represent inferred genotypes based on clinical isolates. Blue circles represent clinical isolates and their known genotypic profile. This figure demonstrates how lineage can be inferred from a genetically diverse set of isolates within a known outbreak. **(B)** Inferred transmission network from the clade 1 southern Nevada outbreak. In this instance we have observed isolates from all intermediates except the most ancestral strain. This representation is derived from the data presented in Table 4.

Distinguishing genetically related isolates within an outbreak can be challenging for pathogens with low rates of mutation (22). A SNP can be the result not only of biological introduction, but also introduced through sequencing and biocomputational methods. Within the outbreak observed herein, core genome assemblies possess a large number of shared SNPs when compared to the CDC clade references and have relatively smaller number of distinguishing mutations that define subgroups (Figure 3A). For example, all but a single clade 1 isolate share 52 common SNPs (Supplemental data), yet Table 4 shows that the most common clade 1 subclade (Group K) differs by only 3 SNPs from the second most common subgroup (Group B). We propose that the usage of subsets of *shared* mutations that follow asexual microbial evolution theory would define the most highly related subgroups (Figure 3A). To this end it can be observed that within a clade, most isolates which share a large number of “core” SNPs compared to the CDC reference, show relatedness relevant to epidemiological investigation. Such cases, then with additional SNPs shared, result in cases with a distinct profile of descendancy and thus would be assumed to have the highest level of relatedness (Table 4).

**Table 4:** Shared SNP analysis of clade 1 isolates.

	SNP Set	1.1	1.2	1.3	1.4	1.5	1.6	1.7	1.8	1.9	1.10	1.11	1.12	1.13	1.14	1.15	1.16	1.17	1.18	1.19	Clade 1 Group
	# SNP in Set	6	1	1	1	1	1	4	3	8	1	1	1	2	3	1	1	1	3	2	
SRR23958479	|	**+**	**+**	**+**	**+**	**+**	**+**	**−**	**−**	**−**	**−**	**−**	**−**	**−**	**−**	**−**	**−**	**−**	**−**	**−**	**A**
SRR23958550	|	**+**	**+**	**+**	**+**	**+**	**+**	**−**	**−**	**−**	**−**	**−**	**−**	**−**	**−**	**−**	**−**	**−**	**−**	**−**	**A**
SRR19664607	|	**+**	**+**	**+**	**+**	**+**	**−**	**−**	**−**	**−**	**−**	**−**	**−**	**−**	**−**	**−**	**−**	**−**	**−**	**−**	**B**
SRR20081625	|	**+**	**+**	**+**	**+**	**+**	**−**	**−**	**−**	**−**	**−**	**−**	**−**	**−**	**−**	**−**	**−**	**−**	**−**	**−**	**B**
SRR23958553	|	**+**	**+**	**+**	**+**	**+**	**−**	**−**	**−**	**−**	**−**	**−**	**−**	**−**	**−**	**−**	**−**	**−**	**−**	**−**	**B**
SRR23958512	|	**+**	**+**	**+**	**+**	**+**	**−**	**−**	**−**	**−**	**−**	**−**	**−**	**−**	**−**	**−**	**−**	**−**	**−**	**−**	**B**
SRR23109092	|	**+**	**+**	**+**	**+**	**+**	**−**	**−**	**−**	**−**	**−**	**−**	**−**	**−**	**−**	**−**	**−**	**−**	**−**	**−**	**B**
SRR19738655	|	**+**	**+**	**+**	**+**	**+**	**−**	**−**	**−**	**−**	**−**	**−**	**−**	**−**	**−**	**−**	**−**	**−**	**−**	**−**	**B**
SRR23958484	|	**+**	**+**	**+**	**+**	**−**	**−**	**+**	**−**	**−**	**−**	**−**	**−**	**−**	**−**	**−**	**−**	**−**	**−**	**−**	**C**
SRR23958472	|	**+**	**+**	**+**	**+**	**−**	**−**	**+**	**−**	**−**	**−**	**−**	**−**	**−**	**−**	**−**	**−**	**−**	**−**	**−**	**C**
SRR23958509	|	**+**	**+**	**+**	**−**	**−**	**−**	**−**	**−**	**−**	**−**	**−**	**−**	**−**	**−**	**−**	**−**	**−**	**−**	**−**	**n/a**
SRR20081622	|	**+**	**+**	**−**	**−**	**−**	**−**	**−**	**+**	**−**	**−**	**−**	**−**	**−**	**−**	**−**	**−**	**−**	**−**	**−**	**D**
SRR20081630	|	**+**	**+**	**−**	**−**	**−**	**−**	**−**	**+**	**−**	**−**	**−**	**−**	**−**	**−**	**−**	**−**	**−**	**−**	**−**	**D**
SRR23958473	|	**+**	**+**	**−**	**−**	**−**	**−**	**−**	**+**	**−**	**−**	**−**	**−**	**−**	**−**	**−**	**−**	**−**	**−**	**−**	**D**
SRR23958536	|	**+**	**+**	**−**	**−**	**−**	**−**	**−**	**−**	**+**	**−**	**−**	**−**	**−**	**−**	**−**	**−**	**−**	**−**	**−**	**E**
SRR19738708	|	**+**	**+**	**−**	**−**	**−**	**−**	**−**	**−**	**+**	**−**	**−**	**−**	**−**	**−**	**−**	**−**	**−**	**−**	**−**	**E**
SRR20081617	|	**+**	**+**	**−**	**−**	**−**	**−**	**−**	**−**	**−**	**+**	**−**	**−**	**−**	**−**	**−**	**−**	**−**	**−**	**−**	**F**
SRR23958480	|	**+**	**+**	**−**	**−**	**−**	**−**	**−**	**−**	**−**	**+**	**−**	**−**	**−**	**−**	**−**	**−**	**−**	**−**	**−**	**F**
SRR23958501	|	**+**	**+**	**−**	**−**	**−**	**−**	**−**	**−**	**−**	**−**	**+**	**−**	**−**	**−**	**−**	**−**	**−**	**−**	**−**	**G**
SRR23958477	|	**+**	**+**	**−**	**−**	**−**	**−**	**−**	**−**	**−**	**−**	**+**	**−**	**−**	**−**	**−**	**−**	**−**	**−**	**−**	**G**
SRR23958478	|	**+**	**+**	**−**	**−**	**−**	**−**	**−**	**−**	**−**	**−**	**−**	**+**	**−**	**−**	**−**	**−**	**−**	**−**	**−**	**H**
SRR19738637	|	**+**	**+**	**−**	**−**	**−**	**−**	**−**	**−**	**−**	**−**	**−**	**+**	**−**	**−**	**−**	**−**	**−**	**−**	**−**	**H**
SRR19738652	|	**+**	**+**	**−**	**−**	**−**	**−**	**−**	**−**	**−**	**−**	**−**	**−**	**+**	**−**	**−**	**−**	**−**	**−**	**−**	**I**
SRR23249014	|	**+**	**+**	**−**	**−**	**−**	**−**	**−**	**−**	**−**	**−**	**−**	**−**	**+**	**−**	**−**	**−**	**−**	**−**	**−**	**I**
SRR23249013	|	**+**	**+**	**−**	**−**	**−**	**−**	**−**	**−**	**−**	**−**	**−**	**−**	**−**	**+**	**−**	**−**	**−**	**−**	**−**	**J**
SRR19738642	|	**+**	**+**	**−**	**−**	**−**	**−**	**−**	**−**	**−**	**−**	**−**	**−**	**−**	**+**	**−**	**−**	**−**	**−**	**−**	**J**
SRR23958528	|	**+**	**+**	**−**	**−**	**−**	**−**	**−**	**−**	**−**	**−**	**−**	**−**	**−**	**−**	**−**	**−**	**−**	**−**	**−**	**K**
SRR23958523	|	**+**	**+**	**−**	**−**	**−**	**−**	**−**	**−**	**−**	**−**	**−**	**−**	**−**	**−**	**−**	**−**	**−**	**−**	**−**	**K**
SRR23958519	|	**+**	**+**	**−**	**−**	**−**	**−**	**−**	**−**	**−**	**−**	**−**	**−**	**−**	**−**	**−**	**−**	**−**	**−**	**−**	**K**
SRR23958514	|	**+**	**+**	**−**	**−**	**−**	**−**	**−**	**−**	**−**	**−**	**−**	**−**	**−**	**−**	**−**	**−**	**−**	**−**	**−**	**K**
SRR23958503	|	**+**	**+**	**−**	**−**	**−**	**−**	**−**	**−**	**−**	**−**	**−**	**−**	**−**	**−**	**−**	**−**	**−**	**−**	**−**	**K**
SRR23958502	|	**+**	**+**	**−**	**−**	**−**	**−**	**−**	**−**	**−**	**−**	**−**	**−**	**−**	**−**	**−**	**−**	**−**	**−**	**−**	**K**
SRR23958500	|	**+**	**+**	**−**	**−**	**−**	**−**	**−**	**−**	**−**	**−**	**−**	**−**	**−**	**−**	**−**	**−**	**−**	**−**	**−**	**K**
SRR23958487	|	**+**	**+**	**−**	**−**	**−**	**−**	**−**	**−**	**−**	**−**	**−**	**−**	**−**	**−**	**−**	**−**	**−**	**−**	**−**	**K**
SRR19664611	|	**+**	**+**	**−**	**−**	**−**	**−**	**−**	**−**	**−**	**−**	**−**	**−**	**−**	**−**	**−**	**−**	**−**	**−**	**−**	**K**
SRR19738706	|	**+**	**+**	**−**	**−**	**−**	**−**	**−**	**−**	**−**	**−**	**−**	**−**	**−**	**−**	**−**	**−**	**−**	**−**	**−**	**K**
SRR19738640	|	**+**	**+**	**−**	**−**	**−**	**−**	**−**	**−**	**−**	**−**	**−**	**−**	**−**	**−**	**−**	**−**	**−**	**−**	**−**	**K**
SRR19738712	|	**+**	**+**	**−**	**−**	**−**	**−**	**−**	**−**	**−**	**−**	**−**	**−**	**−**	**−**	**−**	**−**	**−**	**−**	**−**	**K**
SRR20081637	|	**+**	**+**	**−**	**−**	**−**	**−**	**−**	**−**	**−**	**−**	**−**	**−**	**−**	**−**	**−**	**−**	**−**	**−**	**−**	**K**
SRR23958556	|	**+**	**+**	**−**	**−**	**−**	**−**	**−**	**−**	**−**	**−**	**−**	**−**	**−**	**−**	**−**	**−**	**−**	**−**	**−**	**K**
SRR23958546	|	**+**	**+**	**−**	**−**	**−**	**−**	**−**	**−**	**−**	**−**	**−**	**−**	**−**	**−**	**−**	**−**	**−**	**−**	**−**	**K**
SRR19738698	|	**+**	**+**	**−**	**−**	**−**	**−**	**−**	**−**	**−**	**−**	**−**	**−**	**−**	**−**	**−**	**−**	**−**	**−**	**−**	**K**
SRR19738690	|	**+**	**+**	**−**	**−**	**−**	**−**	**−**	**−**	**−**	**−**	**−**	**−**	**−**	**−**	**−**	**−**	**−**	**−**	**−**	**K**
SRR19738686	|	**+**	**+**	**−**	**−**	**−**	**−**	**−**	**−**	**−**	**−**	**−**	**−**	**−**	**−**	**−**	**−**	**−**	**−**	**−**	**K**
SRR19738668	|	**+**	**+**	**−**	**−**	**−**	**−**	**−**	**−**	**−**	**−**	**−**	**−**	**−**	**−**	**−**	**−**	**−**	**−**	**−**	**K**
SRR23109093	|	**+**	**+**	**−**	**−**	**−**	**−**	**−**	**−**	**−**	**−**	**−**	**−**	**−**	**−**	**−**	**−**	**−**	**−**	**−**	**K**
SRR23109093	|	**+**	**+**	**−**	**−**	**−**	**−**	**−**	**−**	**−**	**−**	**−**	**−**	**−**	**−**	**−**	**−**	**−**	**−**	**−**	**K**
SRR23249015	|	**+**	**+**	**−**	**−**	**−**	**−**	**−**	**−**	**−**	**−**	**−**	**−**	**−**	**−**	**−**	**−**	**−**	**−**	**−**	**K**
SRR19738677	|	**+**	**−**	**−**	**−**	**−**	**−**	**−**	**−**	**−**	**−**	**−**	**−**	**−**	**−**	**+**	**−**	**−**	**−**	**−**	**L**
SRR19738650	|	**+**	**−**	**−**	**−**	**−**	**−**	**−**	**−**	**−**	**−**	**−**	**−**	**−**	**−**	**+**	**−**	**−**	**−**	**−**	**L**
SRR23958539	|	**+**	**−**	**−**	**−**	**−**	**−**	**−**	**−**	**−**	**−**	**−**	**−**	**−**	**−**	**−**	**−**	**−**	**−**	**−**	**n/a**
SRR19738715	|	**−**	**−**	**−**	**−**	**−**	**−**	**−**	**−**	**−**	**−**	**−**	**−**	**−**	**−**	**−**	**+**	**+**	**−**	**+**	**M**
SRR23109090	|	**−**	**−**	**−**	**−**	**−**	**−**	**−**	**−**	**−**	**−**	**−**	**−**	**−**	**−**	**−**	**+**	**+**	**−**	**+**	**M**
SRR23109088	|	**−**	**−**	**−**	**−**	**−**	**−**	**−**	**−**	**−**	**−**	**−**	**−**	**−**	**−**	**−**	**+**	**+**	**−**	**−**	**N**
SRR19738688	|	**−**	**−**	**−**	**−**	**−**	**−**	**−**	**−**	**−**	**−**	**−**	**−**	**−**	**−**	**−**	**+**	**+**	**−**	**−**	**N**
SRR19664610	|	**−**	**−**	**−**	**−**	**−**	**−**	**−**	**−**	**−**	**−**	**−**	**−**	**−**	**−**	**−**	**+**	**−**	**+**	**−**	**O**
SRR23958488	|	**−**	**−**	**−**	**−**	**−**	**−**	**−**	**−**	**−**	**−**	**−**	**−**	**−**	**−**	**−**	**+**	**−**	**+**	**−**	**O**

SNP Set refers to a SNP or set of SNPs that are found only in a subset of strains in this analysis. # SNP in Set refers to the number of SNPs in each SNP set (this ranges from 1 SNP to 8 SNPs). The Clade 1 Group refers to assigned designations of all isolates that have the same shared SNP pattern.

### 3.8. Inference of relatedness among *Candida auris* clade I outbreak samples

In order to utilize whole-genome variation to provide disease control investigators with data regarding the most related sets of isolates, specific SNPs were studied for evidence of inheritance patterns. Clade I (*n* = 60) isolates were analyzed using kSNP3 using the clade I CDC reference strain [strain B11205] as our reference (21). This analysis generated 208 SNPs whereupon the parent (defined as the reference strain) or variant sequence was detected in all 60 strains. Of these SNPs, 109 were present in only one isolate (aka unique SNPs). Of the 99 SNPs that were shared among two or more Clade I isolates, 54 were present in 57 out of 60 clade I isolates. The remainder of the analysis focused upon these 57 strains which all shared this set of “core NV clade I” SNPs.

Among these 57 strains 19 SNP sets were identified which differentiated this group based on genetics. These SNP sets (Table 4) contained between 1 and 8 SNPs. The presence of the SNP set in an isolate is represented with a “+” in Table Y and its absence is represented with a “-“. We identified 15 groups of isolates that had more than one member and had a unique combination of SNP sets (Table 4)—we designated these as clade 1 Groups A through O. Provision of group designations was to assist disease controllers in Nevada in having a nomenclature to describe cases. It was not an attempt to create a novel general nomenclature field-wide. Lastly, we present a subset of our inferred clade 1 transmission network based on these data in Figure 3B.

### 3.9. Inference of relatedness among *Candida auris* clade III outbreak samples

The analysis described above was repeated for the 148 Clade III isolates using the clade III CDC reference strain [strain B11221] as our ancestral outgroup for kSNP3. This analysis generated 401 SNPs where the parent or variant sequence was detected in all 148 strains. Of these SNPs, 280 were present in only one isolate (aka unique SNPs). Of the 121 SNPs that were shared among two or more clade III isolates, 28 were present in 147 out of 148 clade III isolates. Focus was placed upon these 147 strains which all shared this set of “core NV clade III” SNPs.

Among these 147 isolates 42 SNP sets were identified which differentiated this group based on genetics. These SNP sets (Supplementary Table S3) contained between 1 and 5 SNPs. The presence of the SNP set in an isolate is represented with a “+” in Supplementary Table S3 and its absence is represented with a “-“. Groups of isolates (*n* = 35) were identified that had more than one member and had a unique combination of SNP sets (Supplementary Table S3)—these were designated as Clade 3 Groups A through KK.

### 3.10. Discovery of new introductions to *Candida auris* to southern Nevada using shared SNP analysis

The shared SNP analysis described above is performed by the Nevada State Public Health Laboratory on a regular basis since September 2022. During that time, this analysis has identified two novel clade 1 introductions. These novel introductions have a unique set of “core” SNPs that are different from the Southern Nevada “core” SNP signature. To quantify this, we ran kSNP3 with four members of the southern Nevada Clade 1 outbreak with the suspected two novel clade 1 introductions (21). The first novel introduction represented by isolate SRR23137821 has 2,519 SNPs not shared by any of the original clade 1 isolates from this outbreak (Table 5). The second novel introduction represented by isolate SRR23920687 has 87 SNPs not shared by any of the original clade 1 isolates from this outbreak (Table 5). While all three have a small, overlapping set of 10 common SNPs when compared to the CDC clade 1 reference strain, the vast genetic diversity detected by the shared SNP analysis shows that these are new introductions.

**Table 5:** Comparison of SNPs in reference to the CDC Clade 1 reference for two strains from the Nevada outbreak.

Isolate	Number of novel mutations compared to the NV clade 1 core isolates
SRR23137821	2,519
SRR23920687	87

Comparison of SNPs in reference to the CDC Clade 1 reference for two strains from the Nevada outbreak.

## 4. Discussion

*Candida auris* is among the most challenging of healthcare-associated infections (2, 6–11). It combines the ability to persist environmentally with inherent drug resistance and the ability to cause significant morbidity and mortality. As such, public health must bring every tool at its disposal to bear on this threat. Herein, we assess one possible tool for its ability to assist in combatting *C. auris* outbreaks: genomics, in response to multiple, complex outbreaks in Nevada, we sought to generate as much genomic intelligence as possible to better understand the spread of the pathogen. The use of genomics to track and to describe pathogens is certainly not novel. However, its application to *C. auris* outbreaks is relatively new. As a fungal pathogen, *C. auris* has been shown to have a mutation rate much slower than other, healthcare associated agents (23–26). Slower mutation rates may result in less diversity in outbreak populations, thus limiting the ability to distinguish cases within transmission networks. Confronting this, we assessed the genomic diversity of whole genome sequences from numerous isolates associated with outbreaks of clade I and clade III. The observations that were made led to the use not only of quantification of SNP distances, but also the recognition of the genomic locations of SNPs that were shared among cases. Utilization of “shared” SNPs was found to provide power to the use of whole genomics for studying *C. auris* during an outbreak. The concept of shared SNPs allowed rational descriptions and delineations of phylogenetic descendancy. The result was the creation of means to more effectively serve disease controllers and epidemiologists in furtherance of their investigations. While this may not make up for a slow mutation rate associated with the pathogen and thus a lower discriminatory capacity, it does create a means to direct investigators to the most related cases in a rational way. The use of shared SNPs has been applied to pathogen genomics in many instances but to our knowledge, this is the first use of the concept to assess case relatedness within a *C. auris* outbreak (27–29). A previous study applied whole genome sequencing to a small outbreak of *C. auris* within a hospital (30). Therein it was shown that in fact there was considerable genomic diversity between multiple isolates taken from the same patient, and also taken from different patients who were roomed together and were likely transmission pairs (30).

Sequencing and genomic analysis provided real benefits to disease controllers and epidemiologists who were investigating these outbreaks in Nevada. It became readily possible to distinguish between ongoing transmission within facilities versus novel introductions into facilities on the basis of shared SNP descendancy. This triggered different strategies and tactics on a facility-by-facility basis which were applied based upon phylogenetic information rather than from best guesses. Lastly, we demonstrated the shared SNP analysis detected two novel clade 1 introductions from outside of southern Nevada. This early detection allowed our public health responders to attempt to contain these new introductions and prevent their establishment in southern Nevada. This was critical because the greater the number of overlapping and ongoing outbreaks a region is experiencing, the more complicated the role of disease control investigators and epidemiologists becomes.

Because multiple tools exist to assess whole genome sequencing of *C. auris*, the work herein rigorously compared and contrasted three such pipelines. Each performed reliably, though specific differences in genome sizes and time-to-answer were found among the three. Notably, pairwise comparisons of SNP distances between fixed sets of isolates across different pipelines revealed that different pipelines will provide different results. This finding indicates that choice and validation of pipelines is not just a matter of formality. As microbiology and bioinformatics continue to merge, it is critical that when new pipelines are constructed that they are validated against existing tools. An ever-increasing number of pipelines does not serve the field of medicine or public health if the pipelines are not clearly assessed from a quality assurance perspective. Much work lies ahead for standards, consultation and accreditation agencies associated with diagnostic science, as emerging bioinformatic tools require rigorous assessment. Even when they are not used as diagnostic tools, their use as aids to epidemiology and disease control will trigger enormous shifts in work-time and resources, which are often limited in the public health realm.

Comprehensive and rapid sequencing of cases as described herein has just begun to impact the public health intervention aspect of the outbreak. Affected sites with continuous transmission have sought additional interventions, including novel means of chemical disinfection and the use of (PCR) screening tests for incoming patients and employees. Unlike the use of sequencing for other hospital acquired infections (e.g., CRE) the comprehensive use of sequencing as shown herein has laid the groundwork for training and familiarization with the use of genomic sequence intelligence. The intense sequencing has additionally led to a highly sophisticated and detailed description of the outbreaks which has gained the attention of elected public servants in the state who have sought additional resources for approaching the outbreak. Additionally, sequencing and analysis have also provided gravitas to the successful actualization of *C. auris* as a reportable entity in Nevada.

This study possesses unique strengths. It included a large number of isolates, collected prospectively in the course of major outbreaks. The study included analysis of two simultaneous, genomically distinct outbreaks (clade I and clade III), which on the surface resembled a singular outbreak. Additionally, the study compares different tools/pipelines rather than merely showing the construction and functionality of one alone.

This study possesses weaknesses of note. While a high number of isolates associated with a large outbreak were assessed, there are significant gaps in the information that matches epidemiologic data to sequencing data. It is difficult to say with certainty that genomic relatedness ascertained herein is guaranteed to be meaningful from the disease control perspective, without more data. Additionally, not all laboratories or public health jurisdictions could necessarily repeat what was performed herein, as massive resources were necessarily harnessed to generate the granularity of intelligence described.

## Data availability statement

The datasets presented in this study can be found in online repositories. The names of the repository/repositories and accession number(s) can be found in the article/Supplementary material.

## Author contributions

AG generated data, performed analysis, wrote sections of the manuscript, and helped in revisions. FA and MS created bioinformatic pipelines, performed analysis, wrote sections of the manuscript, and helped in revisions. LM generated a figure and helped in revisions. DS, CH, PD, ES, and CN generated data for the paper and helped in revisions. KL and JS supported and funded the creation of bioinformatic pipelines and helped in revisions. SVH provided administrative supervision of the work. MP conceived of and authored portions of the manuscript and provided review. DH conceived of the projects, generated data, performed analysis, wrote the manuscript, and helped in revisions. All authors contributed to the article and approved the submitted version.

## Funding

This research was funded by Centers for Disease Control and Prevention (CDC) Surveillance for Healthcare-Associated and Resistant Pathogens (SHARP); and CDC Epidemiology and Laboratory Capacity (ELC) funding.

## Conflict of interest

Authors FA, MS, KL and JS were employed by Theiagen Consulting LLC.

The remaining authors declare that the research was conducted in the absence of any commercial or financial relationships that could be construed as a potential conflict of interest.

## Publisher’s note

All claims expressed in this article are solely those of the authors and do not necessarily represent those of their affiliated organizations, or those of the publisher, the editors and the reviewers. Any product that may be evaluated in this article, or claim that may be made by its manufacturer, is not guaranteed or endorsed by the publisher.

## Footnotes

^1^ https://benlangmead.github.io/aws-indexes/k2

^2^ https://github.com/CDCgov/mycosnp-nf

^3^ https://github.com/garviz/MUMmer/blob/master/nucmer

^4^ https://github.com/lh3/bwa

^5^ https://github.com/shenwei356/seqkit

^6^ https://github.com/lh3/seqtk

^7^ https://github.com/LANL-Bioinformatics/FaQCs

^8^ https://github.com/samtools/

^9^ https://github.com/broadinstitute/picard

^10^ https://github.com/s-andrews/FastQC

^11^ https://github.com/ewels/MultiQC

^12^ https://github.com/broadinstitute/gatk

^13^ https://gatk.broadinstitute.org/hc/en-us/articles/360035890471-Hard-filtering-germline-short-variants

^14^ https://github.com/samtools/bcftools/releases

^15^ https://github.com/johnlees/rapidnj

^16^ https://github.com/citiususc/veryfasttree

^17^ https://github.com/stamatak/standard-RAxML

^18^ https://github.com/Cibiv/IQ-TREE
