# Supplementary figures and images for: The use of whole-genome sequencing and development of bioinformatics to monitor overlapping outbreaks of Candida auris in southern Nevada
Source: Front Public Health. 2023 Jul 13;11:1198189. doi: 10.3389/fpubh.2023.1198189 (PMC10374848; doi:10.3389/fpubh.2023.1198189)

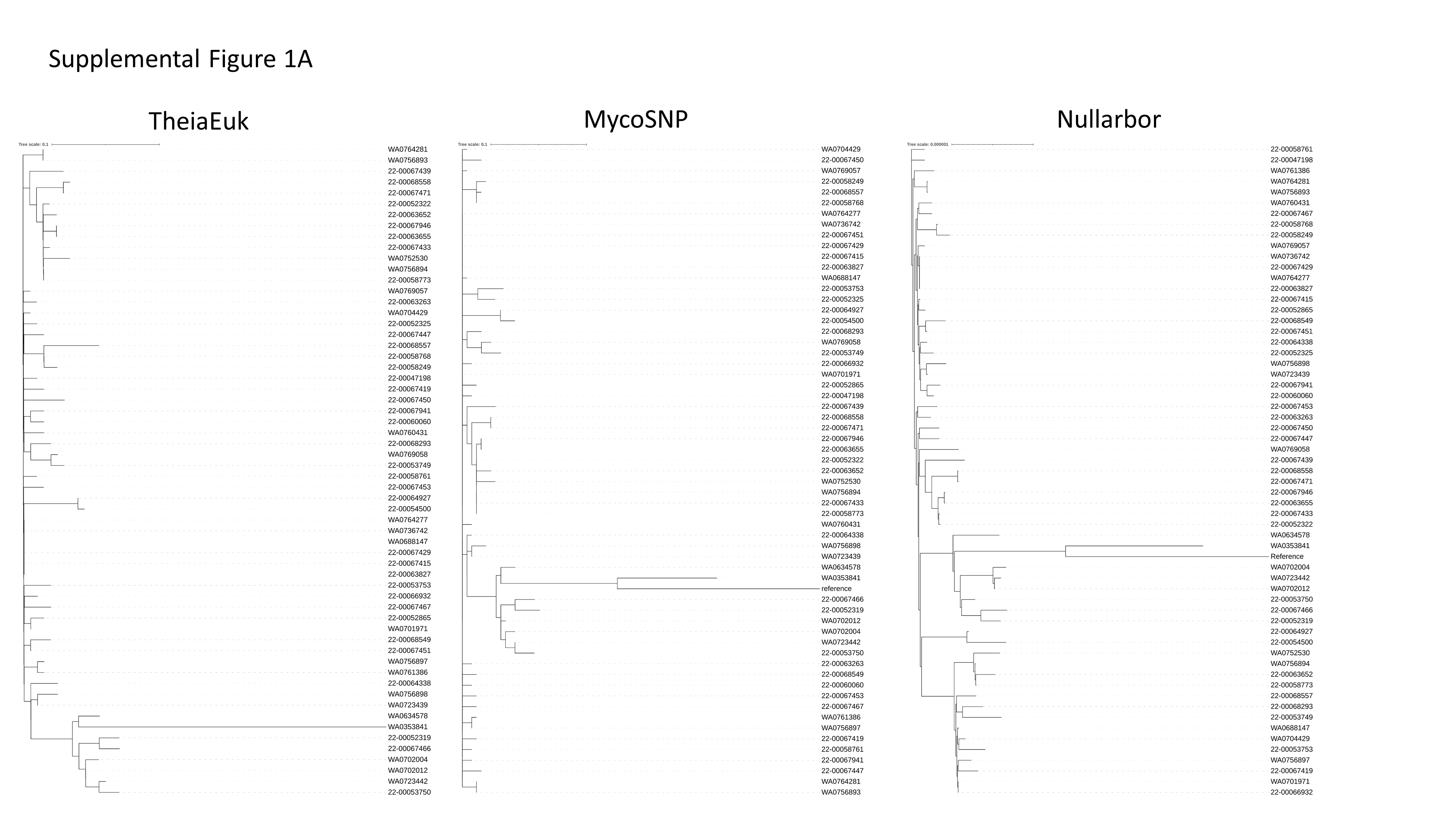

Supplement: Supplementary file 5 [file Image_1.TIF]

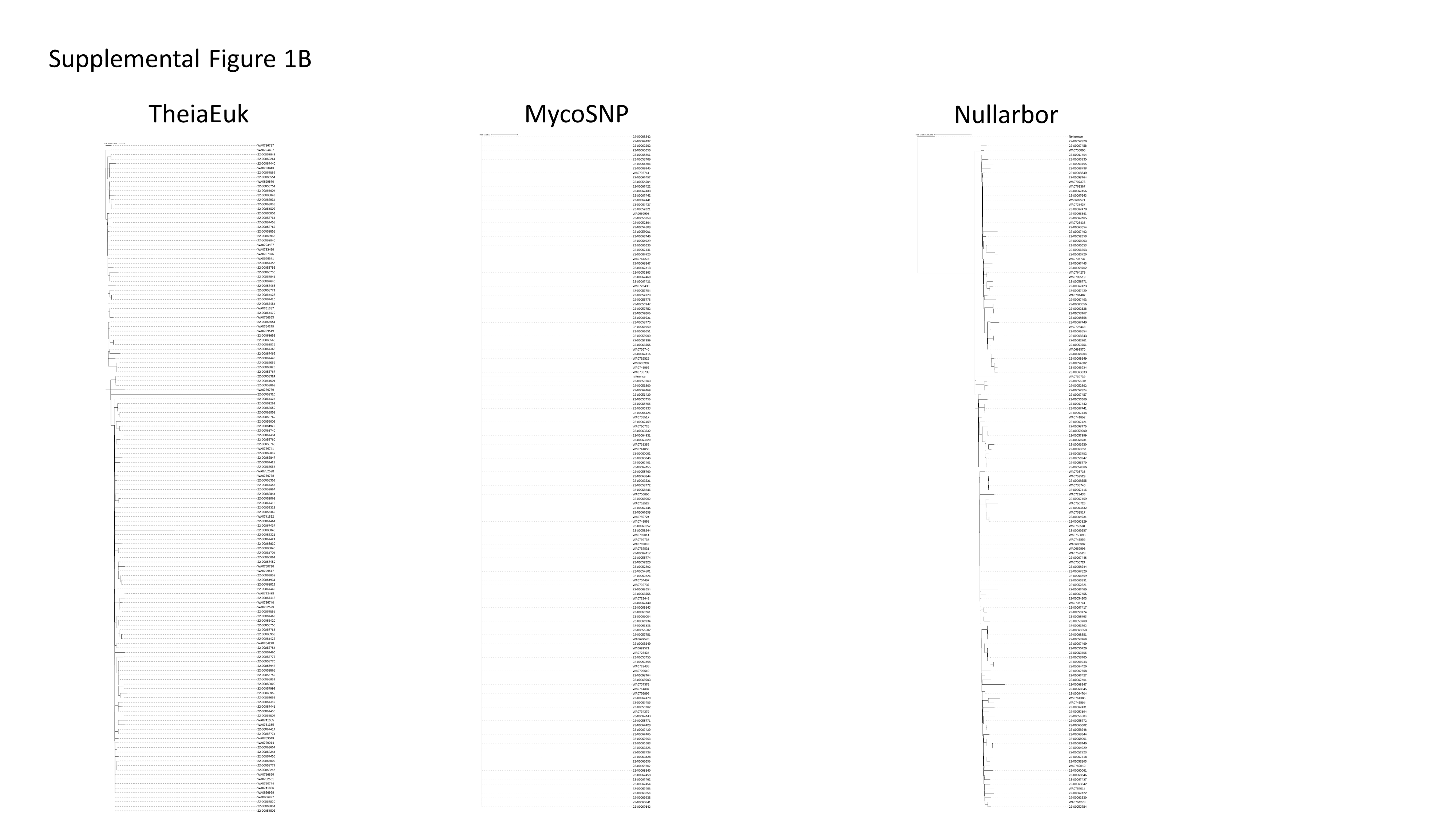

Supplement: Supplementary file 6 [file Image_2.TIF]
